# A review on conductive polymeric nanocomposites for advanced electromagnetic interference shielding: materials, mechanisms, and applications

**DOI:** 10.1039/d6ra03128e

**Published:** 2026-05-26

**Authors:** Akram Fallah, Narges Elmi Fard, Donya Aliyari, Reza Ghamarpoor, Bahram Ramezanzadeh

**Affiliations:** a Department of Chemical Technologies, Iranian Research Organization for Science and Technology (IROST) Tehran Iran; b Department of Chemistry, SR.C., Islamic Azad University Tehran Iran; c Department of Surface Coatings and Corrosion, Institute for Color Science and Technology Tehran 16765-654 Iran; d Department of Petroleum Engineering, Faculty of Engineering, University of Garmsar Garmsar Iran rezaghamarpoor@fmgarmsar.ac.ir +98-23-31930330

## Abstract

The increasing complexity and density of electronic systems have amplified the need for effective electromagnetic interference (EMI) shielding materials to ensure functionality, signal integrity, and compliance with electromagnetic compatibility regulations. Conventional shielding materials such as metals suffer from inherent drawbacks including high density, rigidity, corrosion susceptibility, and processing difficulties. In this review, conductive polymeric nanocomposites have been evaluated as a highly promising group of materials for use as EMI shielding due to their distinctive blending of lightweight nature, flexibility, processability, and tuneful electrical characteristics. These nanocomposites are typically composed of insulating matrix of polymer embedded with electrical-conductive nano-fillers like carbon nanotubes (CNTs), carbon black, graphene or metallic nanoparticles. The incorporation of these fillers enables the formation of percolative conductive networks inside the polymer matrix, which significantly enhances the electrical-conductivity and EMI shielding effectiveness (SE) of the obtained material. Shielding in these materials arises from reflection and absorption of incident electromagnetic waves, with multiple internal reflections contributing under conditions of relatively low absorption. The scalability and environmental resistance of conductive polymeric nanocomposites make them attractive for use in industries such as aerospace, automotive, consumer electronics, and healthcare, where multifunctional, lightweight, and durable materials are essential. In conclusion, conductive polymeric nanocomposites show a versatile and future-ready solution for EMI shielding, bridging the gap between performance, functionality, and manufacturability. Their adaptability and technological advancement make them central to next-generation electromagnetic shielding materials. Surface functionalization of fillers enhances compatibility with the polymer matrix, promoting uniform dispersion and improved interfacial polarization, contributing to absorption-dominant shielding.

## Abstract 2

Rising complexity in electronic systems makes effective EMI shielding essential for performance and compliance.

## Graphical abstract

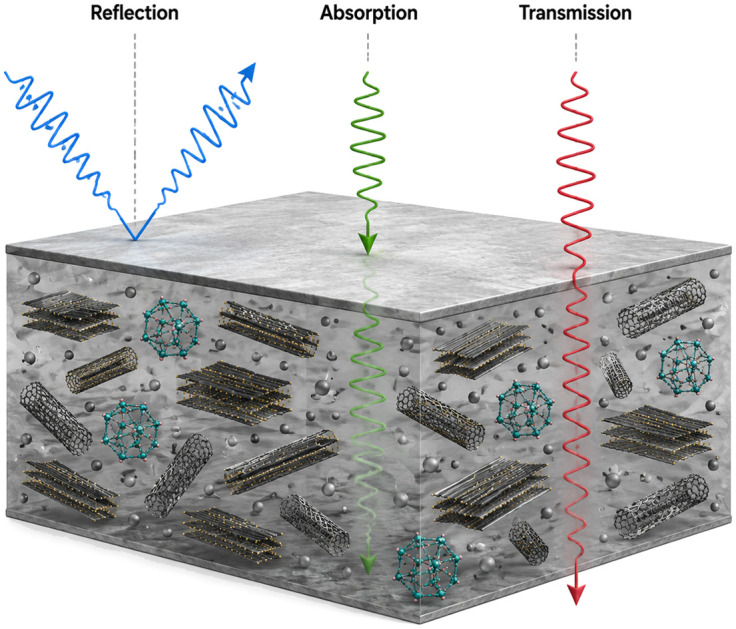

## 1. Introduction

The evolution of technology has caused the release of various pollutants in societies, posing a threat to human health and the environment.^1,2^ Electromagnetic (EM) wave pollution is growing due to increasing usage of communication systems and electronics devices generating EM waves during the operation.^3^ Intelligent electronic machines, remote controls, towers of 4G and 5G cells, broadcast towers of TV and radio, power lines, radar systems, and satellites are some of the EM waves resources in the environment from earth and ocean to space.^4^ Ensuring safety of electrical devices and healthcare are critical areas to provide regular performance of EM systems through developing smart compounds for shrinking, absorbing, and removing of EM waves interferences.^5^

Material composition plays a determining role in removal of the striking EM waves by three primary mechanistic routes of absorption, reflection, and transition.^6^ Reflection route is attributed to conductive materials with high impedance mismatching among the interface of the shielding material and the surrounding space.^7^ Absorption route of striking EM waves as one of the mechanistic route of EMI attenuation cause temperature elevation and heat generation, which result in destruction of shielding composition and performance deterioration.^8^

Utilizing pure metals like stainless steel, Cu, Ag, Au, and Al was traditional approach to shielding interferences of EM waves.^9^ Excellent EMI shielding effectiveness (EMI SE) is consistent with efficient electrical conductivity of the shielding materials. Reflection is the dominant mechanistic route of EMI shielding by direct utilizing of pure metals, which bring about adverse effects due to secondary reflected waves as succeeding pollutants.^10–12^ Secondary reflected waves are causative fields resulting from free electrons or holes vibration in the surface of the conductive shield due to striking of EM waves.^13^

Thermal conductivity of the pure metals as EMI shielding materials increases with elevation of electrical conductivity, which considered as a drawback of shielding fabrication.^14–16^ High density, heavy weight, corrosion susceptibility, rigidity, and high cost require applying modification techniques to enhance absorption contribution in the mechanistic route of EMI shielding and improve ductility, mechanical strength, easy covering, design flexibility, corrosion resistance.^17,18^

A more recent approach is resulted in fabrication of composite structures with conductive characteristics like conductive polymers, ferromagnetic oxides, some carbon-based materials, carbon nanotubes, MXenes, and multi-functional hybrids through enhancing of EMI SE, decrease in density and thickness of EMI shielding materials, and proper physical-structural properties.^19^ Single-component composites of magnetic and dielectric materials suffer from disability in impedance matching and significant attenuation of striking EMI, simultaneously. Multi-component composites with distinct morphology and structure enhance reflection loss and absorption of striking EMI in the broad frequency bandwidth.^20,21^

Proficiency of EMI shielding compounds to dissipate and attenuate is determined by their distinct structural composition and potential to reflect, transmit, and absorb the incident interference waves.^18,22^ Conductive polymer nano-composites are known as the suitable synthetic alternative of traditional metallic EMI shielding materials. Processability and cost-effectiveness of polymer base materials conduct an inherent approach by facilitated techniques of extrusion and injection molding for industrial performance. Polymer composites with electrical conductivity are prepared by adding conductive fillers into dielectric polymer matrix, which provide a conductive surface network of the fillers.^23–25^ The dominant mechanistic route is absorption by polymeric-based nano-composites to attenuate striking EMI, which is improved by proper combination of fillers in an integrated and continuous network throughout the dielectric matrix of polymer. Balancing between conductivity of fillers and appropriate dielectric-magnetic properties result in efficient EMI dissipation. Excessive amounts of conductive fillers cause secondary reflection, which is considered as a deteriorating effect especially in military and aerospace performance areas. Engineering and design of polymer nano-composites determine the dominant mechanistic route of EMI shielding.^26,27^ Wei *et al.*^28^ reported preparation of integrated silver nano-wires into polymeric matrix that demonstrated high efficiency towards shielding against striking EMI waves among long lengths of wideband frequency from 8 to 40 GHz. The prepared Ag-polymeric composite with 1 mm thickness ensured total shielding effectiveness (SE_T_) up to 11.5 dB.

MXene as a conductive nano-composite with M_*n*_X_*n*+1_T_*x*_ structure demonstrates excellent characteristics as the base of multi-component materials in EMI shielding area due to high specific surface area, extended aspect ratio, and outstanding electronic properties. Integration of nano-carbon sphere, carbon black, r-GO aerogels, and magnetic compounds are reported as coating with significant EMI shielding effectiveness due to striking waves shrinking by absorption routes.^29–31^ Hu *et al.*^32^ prepared Ti_3_C_2_T_*x*_ in a wax matrix and reported its EMI shielding effectiveness up to 20 dB over the wide frequency range of 2–18 GHz. The wax matrix loaded with 70 wt% Ti_3_C_2_T_*x*_, with a sample thickness of approximately 2 mm, was evaluated at 18 GHz and exhibited an EMI SE of 34 dB. Persistence and durability of MXene based materials under vacuum atmosphere are up to 800 °C, which make them appropriate candidates for EMI shielding without structure destruction. Theoretically, a conductive polymeric matrix with separated networks prepared a broadway of transferring electrons with significant electrical conductivity and EMI SE. An abundance of interface reflections result in high absorption loss.^33^ Qian *et al.*^33^ stated preparation of ZnO-MXene nanocomposite with excellent electrical conductivity of Ti_3_C_2_T_*x*_ and integrated with semiconductor ZnO compound. The obtained nono-composite demonstrated EM absorption of striking interference waves with proper reflection loss of −26.30 dB. Optimization of ZnO loading improved absorption approach of EMI in the range of 14–18 GHz.

Porosity of the polymeric matrix decrease in density and weight as EMI shielding materials for infrared stealth, and improve SE of the nano-composites due to moderating thermal conductivity with inhibition of heat diffusion throughout the matrix.^5^ Li *et al.*^34^ claimed that prepared a dual-functionality of CoS_*x*_/MnS/C nanocomposite. Vulcanization of the fabricated nano-composite enhanced their conductivity and in turn conductive loss, as well as presence of CoS_*x*_ ensured magnetic loss of the striking EMI on the surface of the prepared nano-composite. Hollow-rich structure, interface between dielectric-magnetic compounds considered as driving force to reflection loss at 10.72 GHz up to −51.31 dB and augmentation of effective absorption bandwidth of 5.92 GHz at thickness of 2.1 mm. Anti-corrosion properties of the prepared nanocomposites along with EMI shielding properties resulted in applicability under corrosive condition. Tang *et al.*^35^ designed and fabricated a modified nano-composite of polyimide non-woven fabric (PI) loaded with Fe_3_O_4_/Ag, appealing in military area. Prepared nano-composite shielded against striking of low-frequency EMI and resistance through stealth of high temperature infrared. Entering further EM waves into the prepared nano-composite resulted in dissipation with SET up to 77 dB and reflection coefficient of 0.09.

Recent advancements in nano-composite fabrication techniques, including *in situ* polymerization, solution blending, melt mixing, and 3D printing, have improved filler dispersion and matrix-filler interfacial interactions both critical factors influencing the EM shielding behavior.^36,37^ Notably, high aspect ratio fillers like CNTs and graphene can achieve excellent SE at relatively low filler loadings, preserving the mechanical integrity and flexibility of the composite. These developments have enabled the design of nanocomposites with tailored electrical, mechanical, and thermal properties for specific EMI shielding applications across a broad frequency spectrum. While challenges remain particularly in achieving uniform large-scale production, optimizing filler content, and balancing electrical with mechanical performance, ongoing research and development continue to push the constraints of nanocomposite capabilities.^38,39^

In this review, conductive polymeric nanocomposites represent a versatile and forward-looking solution for EMI shielding, bridging the gap between performance, functionality, and manufacturability. Their adaptability and continued technological advancement position them as a cornerstone for the next generation of EM shielding materials.

## 2. Key properties of nanomaterials in EMI shielding

Unique characteristics of nanomaterials are widely conducted to decrease the effects of EM irradiation and protect sensitive electronic devices from destruction. The reason behind their effectiveness in EMI shielding lies in their excellent capability in the absorption and reflection of EM waves; hence, these are the prime necessities in modern shielding applications.^8,40^ The field of EMI shielding has changed significantly since an innovative solution of nanoparticles was introduced. Nanoparticles provide a perfect solution to EMI problems because of their special qualities, which include a significant surface-to-volume ratio, remarkable electrical and thermal conductivity, low weight, and chemical adaptability.^41^ Because of their complex chemical structures and multipurpose qualities, materials like MXene, graphene, MOF, and COF are at the forefront of this technological advancement.^42,43^ These nanoparticles are essential in sectors like electronics, automotive, and aerospace since they not only improve shielding performance but also provide lightweight, flexible, and effective solutions.

High electrical conductivity is one of the striking features of nanomaterials, which reinforces their ability to reflect EM waves in an efficient way.^44–47^ One of the most well-known 2D nanoparticles, MXenes, the typical formula for these materials is M_*n*+1_XT, where T is for surface functional groups like –OH, –O, or –F, X for C or N, and M stands for the transition metal. MXenes' two-dimensional structure contributes to their outstanding electrical conductivity as well as a strong ability to reflect EM radiation.^48^ Furthermore, the MXene surface's functional groups enable it to be easier for waves to be absorbed and transformed into heat, lessening the negative impacts of EM interference (EMI). A further significant nanoparticle with special properties is graphene, which has a two-dimensional carbon hexagonal structure.^49,50^ Another important advantage of nanomaterials, besides conductivity and stability, lies in the potential of these compounds to absorb EM waves and then convert them into heat.^8,51^ Especially, the porous nanomaterials, including MOFs and COFs, show outstanding features in this regard. Their porous structures enhance energy absorption by increasing the interaction among the material and EM waves. In addition, the nanoscale design of these materials enhances their surface-to-volume ratio considerably, enabling a stronger interaction with EM radiation. Hence, such materials give improved performance of shielding based on the combination of reflection and absorption capabilities.^52,53^ The performance of nanomaterials can be improved further by using composite nanoparticles having tunable dielectric and magnetic properties. For example, composite nanoparticles made from metals and ferrite magnetic materials are the most suitable candidates for EMI shielding because of their unusual capability to manipulate EM waves. This is because dielectric and magnetic attributes combined bring about improved wave absorption and reflection, thus making these materials highly versatile for different shielding applications.^54^ Another big plus with nanomaterials is their lightweight, flexible nature, which is an added asset mainly in industries related to aerospace and automotive engineering. Materials like graphene or COFs are naturally light but strong, hence perfectly fit for designing flexible and portable shielding solutions. The capability of designing a material that is light without compromising on the performance of the material becomes very crucial for applications where reducing weight would become very vital, like aircraft or electric vehicles. Moreover, their flexibility makes them capable of being bent into complex shapes and merged with modern designs, further extending their use. The versatility of nanomaterials may be increased using different surface modification techniques that increase their survivability under extreme environmental conditions.^55^ Graphene is characterized by its extremely high electrical conductivity, low weight, and mechanical flexibility. Because of these characteristics, graphene is an ideal material for developing protective materials that are both lightweight and durable. Graphene's chemical structure also allows for chemical modification of its properties, which is particularly valuable in adapting it to specific industrial needs. Moreover, because of its very large surface area, graphene interacts more with EM waves, increasing its ability to absorb and reflect. In this regard, modifying the surface properties of nanoparticles can instill in them potential resistance to corrosion or other environmental factors.^56^ That kind of adaptability ensures the continuity and effectiveness of nanomaterial-based shielding solutions in the most severe conditions. These are strong yet versatile nanoparticle-based materials with diverse characteristics that have commended them to the different applications found in various fields. Moreover, the dielectric and magnetic properties can be adjusted precisely to the application of the material in use, further improving performance and functionality of the materials. With their unique combination of absorption, reflection, and durability, nanomaterials surely play a part in the development of advanced shielding technologies. For example, porous materials, such as MOFs and COFs, use their structure to maximize absorption, while composite nanoparticles with magnetic properties enhance wave manipulation. All these features enable superior EMI shielding performance, which can protect sensitive equipment and systems from harsh environments with high levels of EMI.^57^ In a nutshell, nanomaterials have a strong combination of properties answering the different needs of modern EMI shielding applications. Such capabilities, coupled with other newly developed surface-modification techniques, enable these materials to be tailored for optimal performance in a broad range of environments. With their unrivaled performance and versatility, nanomaterials are paving the way toward the next generation of advanced shielding compounds.

## 3. EMI shielding theory and mechanisms

EMI refers to unwanted EM signals that can disrupt or degrade the performance of electronic devices. These signals originate from any device that generates, transmits, or distributes electrical energy. Both conducted and radiated EMI can be transmitted *via* wires, and to prevent interference from damaging sensitive electronic components, shielding is required.^58^ EMI shielding works by blocking or reducing three primary processes for EM radiation:

• Reflection: the shield material reflects incoming EM waves at its surface. The interaction of reflected EM radiation with holes and electrons causes reflection whenever it incidents a shielding material containing active charge carriers. These shields are predicted with superior conductivity, whereas metals often use reflection as their primary strategy for reducing EM radiation.^59^

• Absorption: the absorption potential of incoming EM waves by shielding materials of is the second step in the shielding mechanism. Some of the waves energy is absorbed by the shielding material, reducing its strength. It is expected that materials getting active magnetic and/or electric dipoles might reduce EM radiation through the absorption process. The energy utilized for the polarization activities is used by these dipoles when they interact with the radiation. As a result, less radiation leaks through the barrier.^60^

• Multiple reflections: the third process, known as multiple total internal reflections, allows materials with layered structures and altered interfaces to contribute to shielding. Certain waves bounce multiple times within the shielding material, gradually losing their intensity before they can cause interference. This process will ultimately aid in improving absorption because the procedure occurs within the shield. When the absorption contribution is greater, the numerous reflections are negligible and can be disregarded. The most often used shielding measurement method is the transmission line waveguide approach, even though there are several methods for evaluating the EMI shielding response of materials, including three procedure of open field, shielded box, and shielded room.^61^

**Fig. 1:**
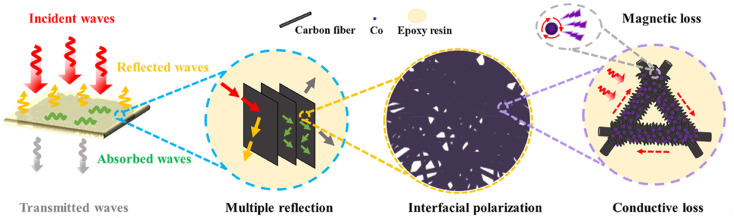
Schematic illustration of the electromagnetic wave attenuation mechanism in the Co/C@TNF-*x*-EP composites. Reproduced from ref. 62 with permission from Elsevier, copyright 2025.

Yin *et al.*^62^ designed a hierarchical epoxy composite based on nickel-plated carbon fiber (NF) felts and *in situ* grown Co-MOF-derived Co/C structures, forming a three-dimensional conductive network within the epoxy matrix. The proposed mechanism (see Fig. 1) indicates that the NF skeleton combined with *in situ* generated Co/C forms a continuous conductive network that enhances conduction loss, while the presence of magnetic Ni and Co nanoparticles introduces magnetic losses such as hysteresis and eddy current effects, collectively leading to efficient attenuation of electromagnetic waves.

The efficiency of EMI shielding is measured in dB and is referred to as shielding effectiveness (SE). A higher SE value indicates better protection against EMI. The SE (eqn (1)) of a shielding material is evaluated by summing up the reflection (SE_R_), absorption (SE_A_), and multiple reflections (SE_M_).^63^

1 SE = SE_R_ + SE_A_ + SE_M_

In practical applications, when SE_A_ > 10 dB, multiple reflections become negligible, and shielding is primarily due to reflection and absorption.

The shielding potential depends on conductivity, magnetic permeability, and permittivity. Materials with high electrical conductivity reflect EM waves, while those with high magnetic permeability absorb them. Thicker shields generally provide better absorption. EM waves penetrate the shield up to a certain depth, known as skin depth (*δ*), which depends on frequency, permeability, and conductivity. The wave intensity decreases exponentially beyond this depth. The skin depth eqn (2) is:^64^2
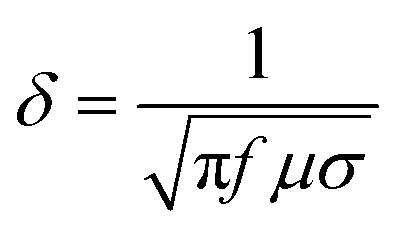
here electrical conductivity is assigned by *σ*, frequency is assigned by *f*, and magnetic permeability is assigned by *µ*.

**Fig. 2:**
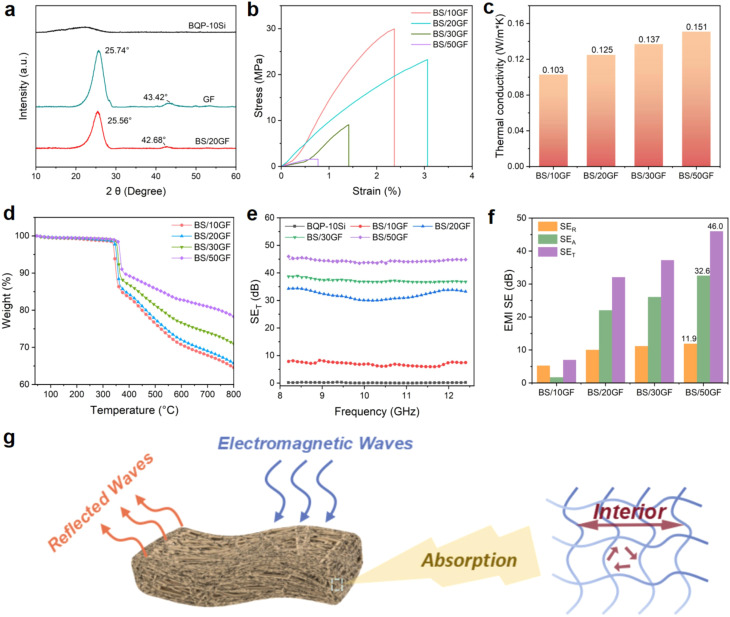
(a) XRD patterns of the polymer and BS/GF composites; (b) stress–strain responses of BS/GF composites; (c) thermal conductivity as a function of GF content; (d) TGA curves of BS/GF composites under nitrogen; (e) SET of BS/GF composites in the X-band; (f) comparison of SER, SEA, and SET values; (g) schematic illustration of the EMI shielding mechanism of the composites. Reproduced from ref. 66 with permission from Elsevier, copyright 2024.

When an EM wave moves between materials with different intrinsic impedance, part of the wave is reflected, and part is transmitted. High conductivity reduces transmission but increases reflection. In the near field (close to the source), EM waves behave differently based on whether they originate from high-voltage/low-current sources (dominated by electric fields) or low-voltage/high-current sources (dominated by magnetic fields).^65^ Wang *et al.*^66^ investigated polyimine/graphite felt (BS/GF) composites and evaluated their structural, mechanical, thermal, and EMI shielding properties. The crystalline characteristics of the polymer and BS/GF composites were analyzed by XRD patterns (see Fig. 2a), while the mechanical performance was assessed through stress–strain curves (see Fig. 2b), showing the reinforcement effect of graphite felt within the polymer matrix. The influence of GF incorporation on heat transport was further examined through thermal conductivity measurements (see Fig. 2c), and the thermal stability of the composites was evaluated by TGA analysis under a nitrogen atmosphere (see Fig. 2d). Regarding electromagnetic performance, the total shielding effectiveness (SET) of BS/GF composites in the X-band (8.2–12.4 GHz) is presented in Fig. 2e, where the SET increases significantly with increasing GF content, reaching about 46 dB for BS/50GF. The individual contributions of reflection (SER) and absorption (SEA) to the shielding performance are summarized in Fig. 2f, indicating that absorption dominates over reflection. The proposed EMI shielding mechanism of the composites is schematically illustrated in Fig. 2g, where conductive graphite fibers promote multiple reflections and absorption of electromagnetic waves, effectively attenuating EMI.

### 3.1. EMI shielding techniques

The level of EMI shielding may be measured using a diversity of techniques. Depending on the sample type, each of these approaches offers advantages and disadvantages from which we may choose the most suitable one. The test techniques listed below are often employed to evaluate a shielding material's EMI SE.^67^

### 3.2. Open field method

The useful shielding efficacy of an entire electronic assembly is assessed by applying the free space or open field method. Therefore, the radiation emissions that leak out of a final product are measured by this test. This technique is used to assess EMI in samples with unique designs or huge dimensions.^68^ The Open Field Method has several advantages over other EMI testing techniques, including being able to test this equipment on a real scale, the ability to more accurately replicate the behavior of EM waves in reality, greater suitability for a broad range of frequencies, particularly high frequencies, and the absence of barriers.^69^ The measurement findings will be far more precise since the radiation use is wave-free. However, drawbacks including external noise like radio waves or power line interference, as well as factors like wind, rain, and humidity, might influence the outcomes. The examination doesn't evaluate whether any a certain material and is prone to significant variances because of changes to the way the various final products are assembled. This is an actual test of the final product's intended shielding service performance. The test procedure entails positioning the apparatus 30 meters away through a receiving antenna and measuring the radiated emissions. The emissions that are sent through the power line are also conducted in the same test.^70^

### 3.3. Shielded box method

The method of shielded box is a popular way to compare test species composed of different shield compounds. This method is often used for small to medium-sized samples which require a safe and isolated laboratory.^13^ The results are not impacted by external signals since ambient noise may have an impact on this test. Additionally, it is economical and perfect for electrical modules and tiny devices. However, the accuracy may also drop at low frequencies because of the limited area. Additionally, the Shielded Box approach eliminates external waves, making it hard to conduct examines that need interaction with ambient signals. The test is set up by a metal box with a sample port in a single electrically tight seam and considered as an antenna for receiving placed on the wall.^71^ A test specimen is installed over the transmitting antenna port that is set outside the box to measure the magnitude of the signals the antenna receives. This method's disadvantage is that it might be challenging to establish sufficient electrical interaction within the shielded box and the examination samples. The other issue is that it only has a 500 MHz frequency range. The findings from several labs indicate.^72^

### 3.4. Shielded room method

The most advanced technique, the shielded room approach, was developed to avoid the shielded box method's limitations. This technique is employed to test larger, more sensitive, and more powerful stations that cannot be tested in shielded boxes in a controlled and isolated setting.^73^ This approach allows for the practical evaluation of equipment, and the results are of excellent quality because there is no ambient noise to interfere with the test. The shielded box technique and the measuring system share the same basic idea, with the exception that the signal source, transmitting and receiving signals, recording device, and measuring system are all segregated in different spaces to get rid of any potential interference. Additionally, the sample size is substantially expanded, usually of the order of 25 cm^2^ in area, and the waves are placed in anechoic chambers the size of a room.^73^ The frequency range across which accurate findings may be acquired is substantially expanded, and the data's repeatability is markedly enhanced, in contrast to the shielded box approach.^74^

### 3.5. Method of coaxial transmission line

Currently, the technique of coaxial transmission line is the suggested approach for assessing SE since it avoids the restrictions of the shielded box methodology. The primary benefit of this approach is the comparability of data from various labs. Furthermore, the data may be separated into its transmitted, reflected, and absorbed components utilizing a coaxial transmission line.^75^ Small samples in the shape of doughnuts are used for testing. A modulated signal can be used to make the measurements at particular frequencies. Sweep mode is composed of a spectrum analyzer and tracking generator as the receiver, or, in the alternative, a tuned amplifier, crystal detector, and generator. In the pointy-point mode, the system is initially set up at a particular frequency in the absence of a sample keeper in the line. When the variable resistor is set to its ultimate amount, the signal level is determined. The attenuator is reduced until the same measurement is obtained after the sample keeper has been inserted into the line.^76^ The effectiveness of the specimen's shielding is directly demonstrated through the reduction of the collected signal. The process is repeated at diverse frequencies in order to obtain a range of reactions. Naturally, it takes a long time, frequently many hours, to make a spectrum using this point-by-point process. A tracking generator driven by a spectrum analyzer alters the engine in the sweeping phase. The spectrum analyzer depicts the system's reaction as a single plot on a monitor in a matter of minutes. The dynamic scope of typical coaxial cables may be as much as 80 dB. The equipment is set up in a small torus-shaped cell that has an internal diameter of 50 mm and an external diameter of 125 mm. The contact resistance was measured after the circular of the 1 mm thick samples was cut. There should be less than 0.2 cm between the sample and the holder. The frequency domain of the observations is 0.01 MHz to 1000 MHz. The coaxial transmission line procedure has been accepted as a recognized standard method.^77^

## 4. EMI shielding applications of smart coatings

### 4.1. Thermal field

The advancements in modern communication and wearable technology have created a demand for films that provide both EMI shielding and operative thermal management.^78^ Advanced electronic films exhibiting multifunctional characteristics are of significant interest for the evolution of integrated intelligent wearable electronic systems aimed at personal therapy and health management. In recent times, multifunctional materials have garnered significant interest in the domains of intelligent systems and human-computer interconnection, necessitating the amalgamation of electrical conductivity, capabilities of sensing, and thermal regulation.^79^ Expectant mothers frequently encounter challenges such as EM pollution, arthralgia, mobility impairments, and irregularities in heart rate. The development of specialized multifunctional intelligent wearable technology to mitigate the risks of EM radiation, alleviate edema and joint discomfort, as well as to monitor physical activity and cardiac pulsations is of paramount importance.^80^ Ran *et al.*^81^ have developed a collection films with sandwich-structure utilizing freeze drying-laying-hot compression machinery, exhibiting impressive EMI shielding of 42 dB, thermal conductivity of 3.29 W m^−1^ K^−1^, and flexibility, thereby enhancing the potential for MXene-based composites in high-performance electronic applications.

In a research, The MXene-PAT-conductive polymer composites were synthesized *via* an economical spray coating method and analyzed using various spectroscopic and microscopic techniques. These composites exhibited impressive electrical conductivity, thermal characteristics, and EMI SE, indicating their potential applications in electronic devices and military technology.^82^ While excessive and uncontrolled heat accumulation during EMI shielding may negatively affect material stability and long-term performance, controlled Joule heating can be beneficially utilized in applications such as thermotherapy, wearable heaters, and de-icing systems.^83^

Through the methodology established by Zhang *et al.*^84^ a composite coating comprising carboxylated carbon nanotubes@MXene/cellulose was developed utilizing a dual-layer coating technique, demonstrating significant advancements in Joule heating (154 °C at 4 V), photothermal conversion (105 °C at 4 suns), and EMI shielding (32.62 dB), thereby presenting a viable approach for the creation of high-performance coatings with multi-functionality applicable in future therapeutic electronic devices.

**Fig. 3:**
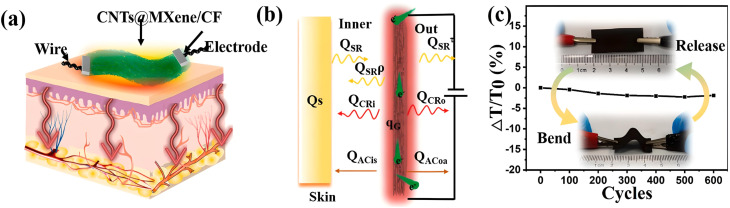
(a) Schematic presentation of a model of electrothermal heat transfer. (b) Illustration of a model of 1D steady-state heat transfer. (c) Functionality of the bending-release cycle dependent temperature, the inset is the images for bending-release analysis. Reproduced from ref. 84 with permission from Elsevier, copyright 2022.

A schematic representation of the electrothermal film is depicted in Fig. 3a. To elucidate the mechanism of continuous heat transfer between CNTs@MXene/CF and human skin, a one-dimensional steady-state heat transfer model is employed (see Fig. 3b), where infrared radiation facilitates beneficial physiotherapeutic effects on the human body. A bending test was conducted to assess mechanical flexibility and structural stability, revealing that after 600 bending cycles, the fluctuation of temperature remains below 2%, indicating the film's remarkable resistance, as shown in Fig. 3c.^84^

**Fig. 4:**
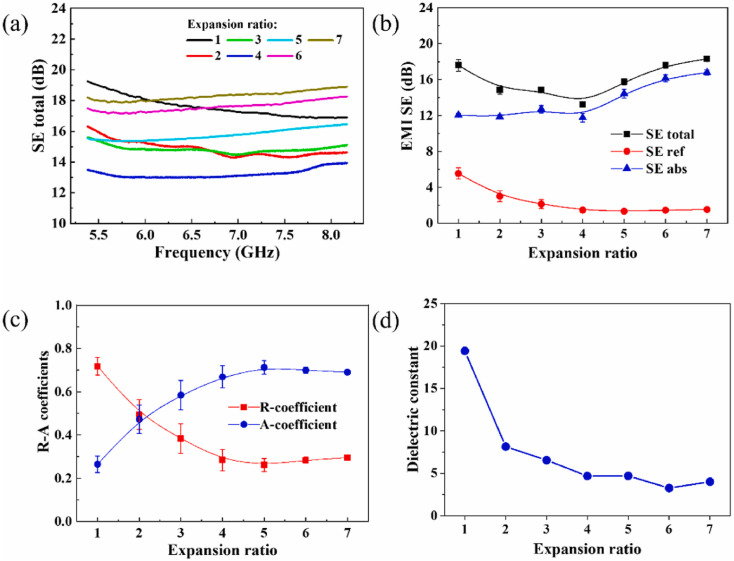
(a) EMI SE spectra of PP/CB (22.5 wt%) composites and their foamed counterparts with different expansion ratios measured in the 5.38–8.17 GHz frequency range; (b) average values of total shielding effectiveness (SE_T_), reflection loss (SE_R_), and absorption loss (SE_A_); (c) average reflection (*R*) and absorption (*A*) coefficients for PP/CB composites and their foams at various expansion ratios; (d) variation of the real permittivity of PP/CB (22.5 wt%) composites and foams near 5.5 GHz. Reproduced from ref. 89 with permission from Elsevier, copyright 2022.

Aerogel materials exhibit significant potential in EMI applications for advanced electronics and weaponry, yet it remains difficult to concurrently optimize self-enhanced shielding efficacy while managing thermal accumulation. Qian *et al.*^85^ have introduced a tunable shielding compound, leveraging synergistic reactions among Ti_3_C_2_T_*x*_ and tungsten-doped VO_2_ as WVO_2_, showcasing superior SE of 42.8 dB and effective thermal management through its intricate three-dimensional structure and phase transitions. Bai *et al.*^86^ propose an innovative approach that integrates an MOF with a textile matrix to achieve EMI shielding, resulting in the development of Co-PCCF from Co@C@carbon fabric (Co-CCF). Co-PCCF can achieve the temperature necessity of thermal therapy through voltage modulation and illumination intensity, while simultaneously enabling assessment of real-time monitoring of diverse human movements such as pulse, respiration, and joint activities. This composite demonstrates exceptional EM shielding capabilities, flexibility, and multifunctionality, making it particularly suitable for pregnant women by mitigating EM exposure and monitoring physiological activities. Wang *et al.*^87^ developed a hydrophobic, multi-responsive film of conductive composite with a core–shell and sandwich structure through two techniques of *in situ* polymerization and dip coating. This 45 µm thick film demonstrates significant conductivity up to 62.15 S cm^−1^, EMI SE of 37.71 dB, alongside excellent waterproof properties, stability in complex environments, and potential application in next-generation wearable electronics. Remarkably, the composite film demonstrates commendable waterproof capabilities, superior electro-optical thermal responsiveness, and robust durability in intricate surrounding environments. Sang *et al.*^88^ developed a wearable electronic device with multifunctional properties by applying conductive MXene onto PVDF film, integrated with PI tape, resulting in a sandwich structure that exhibits superior mechanical strength, tensile toughness, and exceptional mechanosensation abilities for monitoring human movements, along with remarkable Joule heating performance achieving temperatures exceeding 80 °C at 6.5 V. PVDF and PI tapes with insulated high-temperature resistant conduct as shielding layers, ensuring the safety and durability of the PVDF/MXene/PI heater, while the composite demonstrates remarkable EMI SE of 40 dB; thus, the multifunctional PVDF/MXene/PI electronic device presents promising performances in thermotherapy of human, human-machine reciprocity, and EM wave protection. Wu *et al.*^89^ demonstrated that the EMI shielding performance of polypropylene/carbon black (PP/CB, 22.5 wt%) composites can be effectively tuned by introducing a microcellular structure. As shown in Fig. 4a, the shielding effectiveness (SE) of PP/CB foams was measured in the 5.38–8.17 GHz range, where the total SE initially decreased with increasing expansion ratio and then increased again at higher expansion ratios. The corresponding contributions of reflection and absorption losses (SE_R_ and SE_A_) are presented in Fig. 4b, indicating that absorption gradually becomes more significant. The variation of reflection and absorption coefficients with expansion ratio is further illustrated in Fig. 4c, revealing a transition from a reflection-dominated mechanism to an absorption-dominated one. Moreover, the change in real permittivity with expansion ratio (see Fig. 4d) confirms that the introduction of microcells partially disrupts the conductive network, influencing impedance matching and shielding behavior.

Wu *et al.*^90^ electrospun polyacrylonitrile (PAN) nanofiber films are coated with polypyrrole (PPy) through vapor deposition technique, creating a PAN@PPy lattice with continuous 3D conductive, which is further enhanced for EMI shielding by the application of Ti_3_C_2_T_*x*_ nanosheets, resulting in films of PAN@PPy/MXene with 55 µm thick that demonstrate a remarkable EMI SE of 32 dB and an crucial surface-specific SE (SSE/t) of 17 534.5 dB cm^2^ g^−1^ across the range of 8.2 to 12.4 GHz, while temperature obtain up to 170.5 °C under a 4 V input voltage, showcasing rapid, sturdy, and long-lasting Joule heating performance, thus indicating significant potential for smart and wearable device applications.

### 4.2. Mechanical force

As the proliferation of electronic devices accelerates and their quantities expand exponentially by passing one hour, there exists an imperative necessity to innovate next-generation of materials in EMI shielding area. These materials must possess qualities such as flexibility, ultra-thinness, lightweight composition, mechanical robustness, and exceptional efficacy in obstructing EM waves.^91,92^

Liu *et al.*^92^ develop PU/Ti_3_C_2_T_*x*_ nanocomposite coatings characterizing a nacre-like nanostructure that exhibit remarkable flexibility, mechanical characteristics (tensile strength and fracture toughness around 100 MPa, and 3.0 MJ m^−3^, respectively), outstanding electric conductivity (around 2897.4 S cm^−1^), superior EMI SE with thickness-specific SE up to (33 771.92 dB cm^2^ g^−1^), and trace thickness (<10 µm), attributable to a bioinspired design and the strategic selection of PU as the polymer substrate, suggesting potential for enhanced EMI shielding properties through optimized polymer interactions for diverse applications. Yang *et al.*^93^ present an efficient and scalable method for fabricating an adoptable MXene-coated aramid paper (MAP) that exhibits remarkable EMI shielding and outstanding electrical heating capabilities, while the aramid fiber enhances its mechanical strength, maintaining a tensile strength up to 73.6 MPa and a folding toleration of 4156 cycles post selective single/dual MXene coating. The MXene with remarkable electrical conductivity enables the MAP nanocomposite containing 9.0 wt% MXene, to achieve an EMI SE and SE/t of 38 dB and 422.2 dB mm^−1^, respectively. EMI SE of MAP remained stable at 37.4 dB after 5000 bending cycles, along with commendable Joule electric-heating performance (130 °C temperature increase at a 5 V input) and outstanding flame-retardancy, thus offering a viable approach for the mass production of multifunctional flexible EMI shielding materials for upcoming wearable technologies. Chen *et al.*^94^ propose a structure with multiscale optimization approach utilizing a scalable spray-coating technique to produce a conductive and transparent Ag nanowire (AgNW) films that exhibit superior EMI shielding and light transmittance, significantly enhancing the connectivity and uniformity of the AgNW lattice through the application of a Ti_3_C_2_T_*x*_ coating which facilitates the welding of nanowire junctions. The film of Ti_3_C_2_T_*x*_-welded AgNW, exhibiting superior EMI shielding (34 dB) and enhanced mechanical stability compared to neat film of AgNW (21 dB), achieves a remarkable EMI SE equal to 49.2 dB with up to 83% irradiation transmittance due to its layered structure, and, when combined with a PET substrate, enables flexible integration of sound monitoring capabilities, indicating its potential in next-generation electronic applications. Huang *et al.*^95^ synthesized Fe_3_O_4_ nanofibers through electrospinning and calcination, subsequently fabricating a composite of Ti_3_C_2_T_*x*_/Fe_3_O_4_/carbon fiber fabric/waterborne PU that exhibits exceptional EMI SE, particularly the FMC12.5–5 variant, which, containing 12.5 wt% Fe_3_O_4_ and 5 wt% Ti_3_C_2_T_*x*_, achieves an impressive SE of 43.6 dB at a trace thickness of 0.4 mm. Their study presents a composite material with remarkable anisotropic thermal conductivity of 0.46 W m^−1^ K^−1^, superior mechanical characteristics (Young's modulus and tensile strength of 113.5 MPa and 15.7 MPa, respectively), flexibility for repeated deformation, and effective electrothermal conversion, achieving 106 °C at merely 2.5 V, thereby advancing the field of EMI shielding and electrothermal conversion. Kim *et al.*^96^ present a study demonstrating the development of multifunctional textiles with capabilities for energy ingathering, EMI shielding, flame persistence, and Joule heating through a straightforward integration method that employs vacuum filtration to deposit cross-linked Ti_3_C_2_T_*x*_, PVA, and PAA onto Hanji paper, with thorough analyses verifying the materials' robust cross-linking, structural integrity, and interface stability, thereby enhancing their multifunctional efficacy. The MPP-H textiles demonstrate exceptional power generation capabilities exceeding 60 minutes with a power and energy densities of 102.2 µW cm^−3^ and 31.0 mWh cm^−3^, respectively, when combined with 20 µL of NaCl solution, while achieving an EMI SE of 437.6 dB mm^−1^ in the X-band of 8.2–12.4 GHz and a superior absorption-to-reflection ratio of 4.5, thereby surpassing current EMI shielding materials; their enhanced some properties of thermo-chemical and mechanical, such as flame resistance, rapid Joule heating, durability, and washability, further underscore their multifaceted applicability, with the MPP-H providing diverse functionalities in a singular, resilient textile through a scalable fabrication approach, thus holding significant promise for wearable and mobility applications. Wang *et al.*^97^ synthesized films of extra-thin, lightweight conductive composite with distinctive structural designs using a solution dip-coating method, achieving an impressive tensile strength and an average EMI SE of 93.55 MPa and 32 dB, respectively, thereby underscoring the efficacy of this bio-inspired approach for the development of materials with multi-functionality characteristics.

### 4.3. Magnetic field

Recent investigations have concentrated on developing advanced materials for EMI shielding that enhance performance while reducing costs and ensuring compatibility with various electronic devices. Organic fillers, characterized by significant conductivity and magnetic permeability, are ideal for high-achievement applications, while metal powders like silver, copper, and nickel are prevalent due to their excellent conductivity, albeit with higher costs and integration difficulties.^98^ Conversely, metal oxides such as iron oxide and ferrites, along with polymer inorganic nanocomposites (PINCs) that incorporate nanoparticles, present an economically viable and efficient approach to achieve superior EMI shielding properties.^99^

Katheria *et al.*^100^ synthesized and examined a novel composite from integrating Ti_3_C_2_T_*x*_ and Fe_3_O_4_-*g*-C_3_N_4_ within a matrix of ethyl methacrylate polymer *via* a solution blending technique, which resulted in a composite with a hierarchical architecture. This composite, designated EX50FG50, demonstrated exceptional EMI SE and enhanced thermal conductivity of −36 dB and 0.98 W m^−1^ K^−1^, respectively, underscoring its potential utility in EMI shielding and thermal managing applications across various industries with advanced technology. Zheng *et al.*^101^ highlight the complexities in improving the EMI shielding efficacy of MXene hetero-compounds through structural engineering approaches. They propose a layer-by-layer assembly method to fabricate multilayer MXene/Fe_3_O_4_ composite that demonstrate insignificant electrical resistance, super-hydrophobic properties, and tunable EMI shielding, thus advancing the high-performance compounds design for wearable electronics.

The demand for stretchable and multifunctional heating capabilities in wearable EMI shielding textiles is paramount to address the escalating EM pollution, particularly for vulnerable populations like pregnant women. Dong *et al.*^102^ stated synthesis of stretchable fabrics of MXene-coated thermoplastic PU (TPU) through a straightforward technique of uniaxial pre-stretching and spraying, resulting in a novel wrinkled structure that imparts effective EMI shielding and strain-invariant electrical conductivity. The stretchable film, characterized by minimal MXene loading, demonstrated significant EMI shielding effectiveness and superior thermal properties, thereby presenting a extraordinary approach for the fabrication of wearable fabrics with multi-functionality. Huang *et al.*^103^ claimed synthesis of a Ti_3_C_2_T_*x*_/MWCNT substrate with hexagonal SrFe_12_O_19_ flakes through a pure filtration method, yielding a film with notable electrical conductivity and impressive EMI SE of 438 S cm^−1^ and 62.9 dB, respectively, at just 40 µm thickness. The enhanced magnetic loss and unique characteristics of the magnetized MXene-based films facilitate efficient EM wave absorption, suggesting innovative applications for EMI shielding materials. Guo *et al.*^104^ reported a vacuum filtration technique in two-step to develop flexible and extremely thin nanofiber of cellulose (CNF)-based composite films featuring an asymmetric layered engineering consisting of a CoFe_2_O_4_@MXene/CNF film with insignificant conductivity and a AgNWs/CNF layer with significant conductivity. The resulting films demonstrate exceptional EMI shielding effectiveness, mechanical robustness, and thermal management capabilities, thus presenting significant applicability across various high-tech sectors, including aerospace and defense. Zhang *et al.*^105^ developed Co–CoFe_2_O_4_@mesoporous hollow carbon spheres (PCHMs) through a two-step process involving the synthesis of 3D mesoporous hollow carbon nanospheres and the *in situ* growing of Co–CoFe_2_O_4_ nanoparticles. The resulting nanocomposites exhibited stable structures, tunable EM properties, and significant potential for absorption of EM wave, demonstrated by a trace reflection loss of −65.31 dB at 2.1 mm and an effective absorption bandwidth of 8.48 GHz.

The performance of polymeric nanocomposites for EMI shielding strongly depends on the type of conductive filler and its interaction with the polymer matrix. MXene-based fillers provide excellent electrical conductivity and layered structures that enhance absorption-dominated shielding, although their oxidation sensitivity may limit long-term stability. Carbon-based fillers such as CNTs and graphene offer high aspect ratios, good mechanical reinforcement, and relatively low percolation thresholds, but uniform dispersion remains a key challenge. Metal nanoparticles and nanowires exhibit extremely high conductivity and are effective for reflection-dominated shielding, yet issues such as oxidation, cost, and weight may restrict their practical use. In contrast, MOF/COF-derived materials introduce high porosity and interfacial polarization sites that favor absorption mechanisms, although they often require hybridization with conductive fillers to achieve sufficient conductivity. Therefore, hybrid nanocomposite systems combining different fillers appear to provide a balanced strategy for achieving high shielding performance, structural stability, and scalable fabrication.

## 5. Polymer composite as EMI shielding

As the intelligent era progresses swiftly, intelligent EMI shielding devices are garnering increasing focus due to their benefits in adaptive environmental responses. Consequently, the selection of suitable EMI shielding materials is essential for obstructing detrimental EM radiation while allowing the passage of functional EM waves.^106,107^ Advanced and smart EMI shielding compounds, which possess the capability to dynamically modify their EMI SE in accordance with specific performance demands and environmental fluctuations, hold significant advantages in both military and civilian sectors. To this point, various materials exhibiting adoptable EMI SE for diverse responses have been developed.^41,106^ This review particularly emphasizes smart materials characterized by tunable EMI SE and concludes by addressing the challenges and future perspectives associated with smart EMI shielding materials.

Conductive polymer composites have progressively supplanted conventional metallic protective materials, largely attributable to their numerous advantageous characteristics, which considered but are not restricted to low density, elevated strength, consistently stable chemical properties, and the inherent ease of processing involved in their production and application.^108,109^ The attainment of relatively satisfactory performance levels in EMI shielding materials can be achieved merely through the incorporation of highly conductive or magnetic fillers, which serve to enhance the material's protective capabilities.^110^ Nonetheless, this approach necessitates the utilization of a substantial quantity of filler, which inevitably results in complications during the processing phase and may result in a compromise in the mechanical characteristics of the resultant composites; furthermore, it poses significant challenges in effectively harnessing the shielding effectiveness (SE) to realize superior protective functionalities.^111^ In an effort to ameliorate the shielding effectiveness in the context of EMI for polymer composites, researchers have endeavored to enhance the performance by employing the parallel filler content while manipulating the surface characteristics of the filler materials or introducing external induction methods; however, the degree of improvement observed remains constrained and is significantly below the anticipated levels of enhancement.^100^

### 5.1. Films

MXene-based elastomeric composites exhibit significant promise for enhancing the functionality of stretchable and wearable electronics, yet developing composites that excel in EM radiation shielding and other multifunctional properties poses considerable challenges. Das *et al.* successfully synthesized composites of ZnO cross-linked and 1-(3-aminopropyl) imidazole (API)-integrated XNBR/MXene, achieving remarkable shielding effectiveness, thermal and mechanical properties, self-healing capabilities, and recyclability, while maintaining structural integrity under various conditions.^112^

**Fig. 5:**
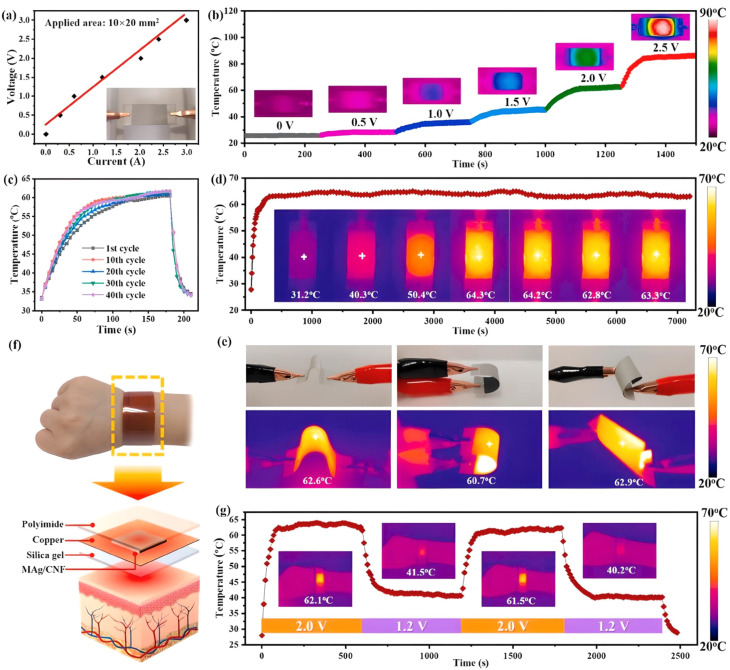
(a) Curves (*I*–*V*) of paper from MAg/CNF composite; (b) temperature curve under variant input voltages from 0 to 2.5 V; (c) temperature adoptability under 40 heating cycles; (d) time–temperature curve at 2 V; (e) digital and corresponding IR-thermal images through diverse bending state at 2 V; (f) schematic demonstration for the thermotherapy potential; (g) the working temperature curve of the thermotherapy instrument and the corresponding images of IR thermal. Reproduced from ref. 114 with permission from Elsevier, copyright 2022.

MXene-based composites represent a viable solution for mitigating EMI due to escalating concerns in this domain. Sha *et al.*^113^ introduces lightweight, ultra-flexible films of PE@PET/MXene with enhanced mechanical strength and EMI shielding efficiency, achieved through innovative fabrication techniques and polydopamine modification. The MAg/CNF composite papers developed by Liang *et al.*^114^ demonstrate significant electrical disparities, obtaining an EMI SE of 82 dB throughout the X-band, while exhibiting stability under extreme temperatures and repeated mechanical stress, thus indicating their applicability for advanced performance in EMI shielding and thermal regulation. The performance and potential applications of the MAg/CNF composite paper were systematically evaluated through a series of experiments, as illustrated in Fig. 5. As demonstrated, Fig. 5a presents the analytical findings and linear regression of the current (*I*) and voltage (*V*) in papers of MAg/CNF composite. The nearly linear *I*–*V* relationship enables precise and controllable thermal regulation. As illustrated in Fig. 5b, the thermal output from the composite papers increases with voltage, aligning with theoretical electric heater models. The surface temperature quickly escalates to approximately 86 °C at 2.5 V, which is significantly lower than that of other conductive materials. Stability assessments through 40 on/off cycles at 2 V reveal consistent temperature maintenance around 62 °C, demonstrating reliability. The composite's exceptional heating performance, even under deformation, suggests substantial potential for applications in personal thermal management devices. To assess thermal reliability, the composite paper was subjected to 40 consecutive heating cycles, demonstrating stable temperature behavior over repeated use (see Fig. 5c). Additionally, a time–temperature curve at a constant voltage of 2 V was recorded, showing rapid heating and stable thermal performance, which illustrated in Fig. 5d. The flexibility of the material was evaluated by bending the composite paper at various angles while applying a 2 V input, with both digital and IR images confirming consistent thermal performance under mechanical deformation (see Fig. 5e). A schematic illustration in Fig. 5f was provided to demonstrate the envisioned use of the composite in thermotherapy applications. Finally, according to Fig. 5g, the practical integration of the MAg/CNF composite paper into a wearable thermotherapy device was validated by monitoring its working temperature profile and visualizing heat distribution *via* IR imaging.

**Fig. 6:**
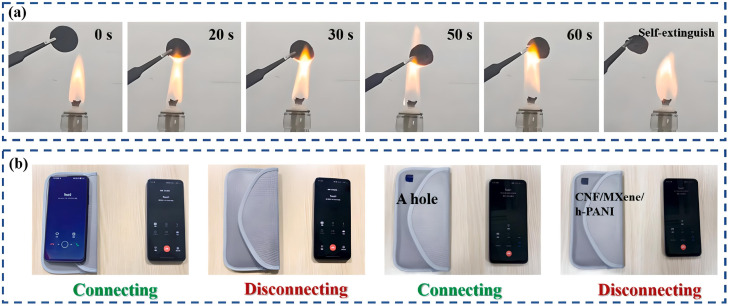
(a) Images of composite film (S-40) after burning during 60 s, (b) an EMI shielding application assessment in shielding bag of cellphone signal. Reproduced from ref. 116 with permission from Elsevier, copyright 2023.

Li *et al.*^115^ present innovative composites with adoptable and self-healing EMI shielding properties, engineered by creating a 3D structure of highly conductive porous Ti_3_C_2_T_*x*_/rGO hybrid aerogel through two main techniques of freeze–drying and chemical reduction, followed by the incorporation of dynamic crosslinked polyurethane with Diels–Alder bonds *via* vacuum-assisted impregnation; these composites of 3D MXene/rGO/PUDA exhibit an significant EMI SE of 39.1 dB at minimal MXene and rGO volumes, demonstrate significant recovery in EMI shielding capability post-damage, and maintain performance stability after extensive mechanical stress, indicating substantial promise for the protection of portable electronic communication devices. Chu *et al.*^116^ developed composite with sandwich structure of EMI shielding film utilizing an alternating vacuum-assisted filtration technique, comprising h-PANI/CNF in the outer layers and MXene/CNF in the layer interface. Their innovative structure obtained an EMI SE of 35.3 dB at distinct compositions, demonstrating remarkable stability against various treatments and a significant 325% increase in absorption coefficient compared with pure CNF film, indicating promising applications in advanced technological fields. The shielding materials are essential for safeguarding electronic devices from accidental leakage or the aging-related fire hazards. Combustion experiments demonstrated that the CNF/MXene/h-PANI composite film exhibits remarkable fire resistance, maintaining structural integrity post-exposure. The composite membrane's effectiveness in blocking signals in compromised EM shielding scenarios suggests significant potential for its application in EM shielding technologies. The fire resistance and EMI shielding capabilities of the S-40 composite film were further demonstrated through practical evaluations, as shown in Fig. 6. An image taken after exposing the composite film to an open flame for 60 s highlights its excellent flame-retardant properties, with minimal structural degradation observed (see Fig. 6a). To assess its real-world applicability in EMI shielding, the composite was tested in a mobile phone signal shielding bag, where it effectively blocked incoming signals, confirming its potential use in practical EMI shielding applications (see Fig. 6b).

In the research conducted by Fei *et al.*^117^ composite films featuring a multilayer sandwich architecture were effectively synthesized from solutions containing cellulose in ferric (MIL-88B) as MOFs-derived magnetic carbon composites (C-MIL-88B) integrated with GNPs through a self-assembly technique, which assisted by filtration. The resulting films of C-MIL-88B/GNP exhibited commendable and significant saturation magnetization and conductivity, achieving an EMI SE of 28 dB throughout the X-band frequency, attributed to the synergistic effects of dielectric and magnetic losses alongside the multilayer configuration, thereby underscoring the promise of MOFs/GNP composites in EM wave absorption applications. Li *et al.*^118^ have developed conductive fabrics of PANI/MXene/cotton (PMCFs) utilizing an innovative technique of vacuum filtration-assisted spray-coating, leveraging the superior MXene conductivity and the acid/basic doping capabilities of PANI for adaptable EMI shielding. The PMCF demonstrates a notable ammonia sensitivity of 19.6% at 200 ppm, achieving an EMI shielding efficiency of approximately 54 dB, while also functioning as a responsive “switch” for varying shielding effectiveness influenced by hydrogen chloride and ammonia vapors, indicating applicability in intelligent textiles and adoptable electronic systems.

Gao *et al.*^119^ developed flexible films of multilayered MXene/thermoplastic PU utilizing a direct method of layer-by-layer spraying, achieving remarkable conductivity up to 1600 S m^−1^, EMI SE up to 50.7 dB throughout the X-band, an outstanding SE of 7276 dB cm^2^ g^−1^, elevated in-plane thermal conductivity and low cross-plane thermal conductivity of 6.31 W m^−1^ k^−1^ and 0.42 W m^−1^ k^−1^, respectively, coupled with impressive Joule heating performance (113 °C) at 5 V, rapid response time, exceptional heating resistance, well organized de-icing, and capability for thermal stealth applications. Liu *et al.*^120^ have innovatively developed a freestanding, ultrathin composite film of Ti_3_C_2_T_*x*_/PEDOT:PSS with a biomimetic “brick-and-mortar” architecture through a vacuum-assisted filtration method. This composite, measuring merely 11.1 µm in thickness, showcases exceptional conductivity and EMI shielding efficiency, highlighting its significant performance in lightweight and adoptable EMI shielding materials. Bian *et al.*^121^ developed multifunctional adopt composite films exhibiting remarkable EMI shielding and strain sensing capabilities *via* a tandem techniques of simple vacuum-assisted filtration and transferring.^121^ These films, featuring ultrathin AgNW/MXene conductive networks on a flexible PDMS substrate, demonstrated exceptional EMI shielding effectiveness and mechanical stability while also exhibiting significant potential for wearable sensor applications in motion and voice recognition. Xiang *et al.*^122^ developed EMI shielding composites utilizing FMs, PET MPs, and MXene through polydopamine (PDA) adhesion, investigating the influence of isolated conductive networks, PET MPs sizes, and FMs layers on the adsorption and electrical characteristics of PP-PDA-MXene composites, which achieved an EMI SE of 30.5 dB throughout the X-band frequency with 1.5 vol% MXene, thereby addressing microplastic recycling post-water removal.

Drawing inspiration from the remarkable stretchability of kirigami designs, Chen *et al.*^123^ introduce a novel bottom-up technique to fabricate conductive and stretchable films of PDMS/Ti_3_C_2_T_*x*_ for innovative multi-application as EMI shielding and pressure sensing, involving the creation of hierarchical wrinkled MXene patterns on an adoptable PDMS substrate, which, through the generation of self-controlled microcracks during prestretching/releasing cycles, grants the films 100% stretchability, strain-invariant conductivity across a 0–100% strain range, and consistent conductivities over 1000 fatigue cycles; the films demonstrate consistence EMI shielding of around 30 dB at 50% tensile strain, improving to 103 dB with a dual-film configuration, while a highly sensitive iontronic sensor array is produced *via* stencil printing, showcasing a sensitivity up to 66.3 nF kPa^−1^, significant dynamic constancy up to 1000 cycles across various frequencies, and effectual pressure surveillance at 50% tensile strain. Ma *et al.*^124^ present an innovative Janus bacterial cellulose (BC)/MXene film produced *via* a simple vacuum filtration technique, demonstrating its skin-contactable potential. Their study highlights the structural integrity and superior properties of the composite, including significant tensile strength and shielding effectiveness, indicating its applicability in wearable technology and military applications.

Film-based conductive composites provide an effective strategy for lightweight and flexible EMI shielding materials. Structural designs such as multilayer architectures and hybrid conductive networks enhance electromagnetic attenuation while maintaining mechanical flexibility. In addition to shielding, many of these films exhibit multifunctional properties such as sensing, thermal management, and self-healing, making them promising for wearable electronics and advanced electromagnetic protection systems.

### 5.2. Foam

The incorporation of MXene into natural rubber latex has resulted in a foam composite that significantly enhances tensile strength by 171% and 157% for 2 and 3 phr MXene, respectively, while also improving electrical conductivity and EM shielding, thus broadening its applicability in industries such as automotive and aviation.^125^ Materials with lightweight EMI shielding properties and impact-absorbing potential are essential for safeguarding sensitive components in portable electronics. The composite of MXene/auxetic PU (MX/APU) foam demonstrates notable EMI SE and impact attenuation capabilities, achieving an exclusive EMI SE of 76.2 dB and reducing peak force by 51.4%, thus positioning it as a promising candidate for lightweight applications.^126^

A scalable dip-coating and chemical crosslinking techniques yield lightweight, ultra-flexible, and durable porous composites of crosslinked Ti_3_C_2_ coated polyimide (C-MXene@PI), which exhibit hydrophobicity, oxidation resistance, superb-temperature resistance, and effective using of MXene's conductivity, interfacial polarization with PI, and pores in µm scales. The C-MXene@PI composites full of porosity exhibit remarkable X-band EMI shielding effectiveness (22.5 to 62.5 dB) at distinct densities, outstanding electrothermal performance as flexible heaters, and reliable electromechanical sensing capabilities for wearable applications, indicating their potential for advancement in flexible electronics and smart devices. Moreover, the integration of composite foams onto the human body effectively evaluates their electromechanical sensing capabilities, evidencing their sensitivity and reliability in detecting human movements as wearable sensors.^127^

Wang *et al.*^128^ synthesized few-layered structure of Ti_3_C_2_T_*x*_ through both techniques of sonication and ionic intercalation, subsequently developing a porous 3D hybrid foam of Ti_3_C_2_T_*x*_/C (MCF) *via* two techniques of sol–gel and thermal reduction. The resulting nanocomposites of MCF/epoxy demonstrated EMI shielding capability, particularly at 4.25 wt% (MCF-5), illustrated optimum conductivity of 184 S m^−1^ and enhanced EMI SE of 46 dB, significantly outperforming MCF-0/epoxy nanocomposites, while also exhibiting increased Young's modulus and hardness. The incorporation of foam structures into shielding materials significantly lowers mass density while improving EMI shielding capabilities. Li *et al.*^129^ developed PVDF foams with CNTs and Ti_3_C_2_T_*x*_, achieving notable anisotropic electrical conductivity due to MXene alignment, which ultimately enhanced EMI shielding effectiveness to 65.1 dB through an absorption-dominated mechanism.

**Fig. 7:**
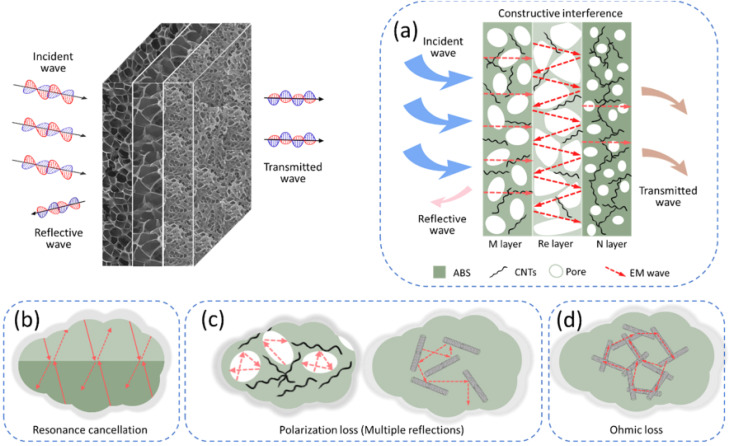
Schematic illustration of the EMI shielding mechanism in three-layered ABS/CNT foams, including (a) constructive interference, (b) resonance cancellation, (c) polarization loss, and (d) ohmic loss. Reproduced from ref. 131 with permission from Elsevier, copyright 2023.

In their research, Li *et al.*^130^ synthesized CoNC@rGO and amine-enriched NH_2_@rGO to create nanocomposites of NH_2_–CoNC@rGO-fluorinated epoxy *via* MW irradiation, achieving a composite that integrates exceptional EMI shielding, high mechanical strength, superamphiphobicity, and enduring characteristics against corrosion symptoms for the first time. The study illuminates the synergistic effects of fluorinated chains and various components on mechanical performance and interfacial bonding, providing a framework for developing advanced epoxy nanocomposites with multifunctional attributes for applications in aerospace and electronic devices. Fu *et al.*^131^ demonstrated that structural design in multilayer ABS/CNT composite foams can effectively tailor EMI shielding toward low reflection and high absorption. In their three-layered M–Re–N architecture, the shielding mechanism was attributed to several synergistic attenuation pathways. As illustrated in Fig. 7a, the sandwich-like arrangement promotes constructive interference of electromagnetic waves, analogous to a Fabry–Perot cavity. Fig. 7b shows that impedance mismatch between adjacent layers induces repeated internal reflections and resonance cancellation, further consuming the incident waves. In addition, the numerous interfaces among pores, CNTs, and the ABS matrix generate polarization loss through multiple reflections and interfacial interactions (see Fig. 7c). Finally, ohmic loss mainly occurs in the nano-porous conductive layer, where the dense CNT network facilitates electron conduction, hopping, and tunneling, thereby enhancing EM wave attenuation (see Fig. 7d). Owing to this rational layer configuration, the ordered three-layer foam achieved the desired combination of reduced reflection and enhanced absorption.

Peng *et al.*^132^ developed multifunctional ultra-light composites, namely CoFe-MOF@Ti_3_C_2_T_*x*_@Na-alginate@WPC (MMSW), utilizing a porous renewable wood-derived carbon framework as a template and microreactors. The MMSW900 exhibits remarkable microwave absorption, flame retardancy, and thermal insulation, resulting a suitable candidate for advanced handling in EMI shielding within the aerospace and defense sectors. Li *et al.*^133^ developed TPU foam (TF) *via* a sacrificial template approach, subsequently fabricating a foam of MXene nanosheet-encased TF (TPU@PDA/MXene) through vacuum-assisted impregnation, utilizing a elastic TF framework with microporous structure and PDA as a binding agent. Their innovative design achieved remarkable conductivity up to 290.8 S m^−1^ and a substantial EMI shielding effectiveness (EMI SE) of 72.2 dB in the X-band, demonstrating a promising and economical method for creating efficient composites as EMI shielding suitable for use in wearable technology and aerospace systems.

**Fig. 8:**
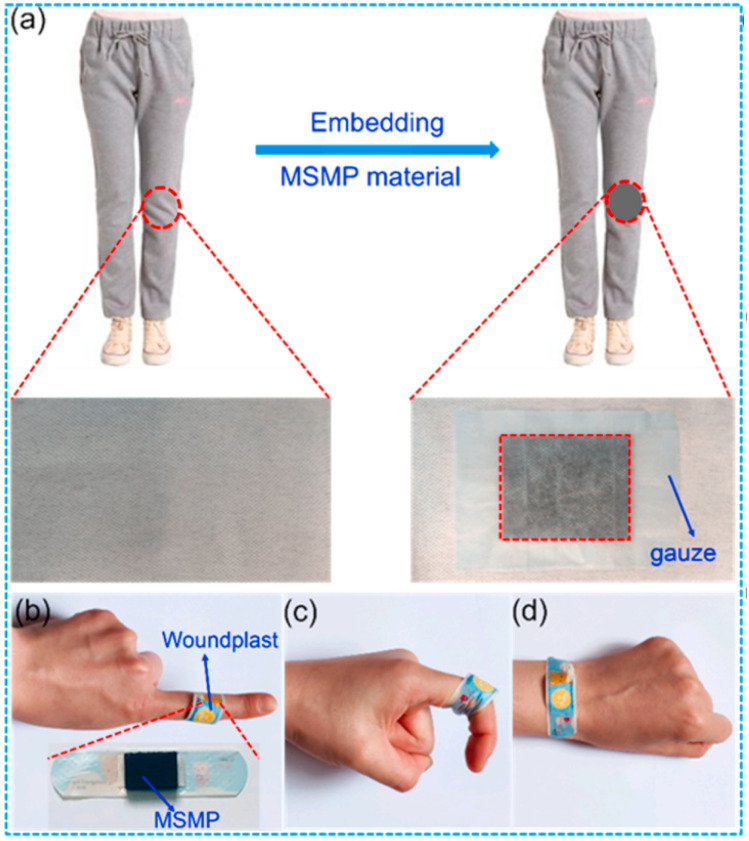
(a) The developed safety sport clothing by embedding MSM57P0.25 composite. (b–d) The wearable resistance characteristics of as-fabricated MSMP composite covered different parts of body. Reproduced from ref. 135 with permission from Elsevier, copyright 2021.

The urgent need for advanced materials as EMI shielding with exceptional SE and absorption capabilities is highlighted in recent research. Guo *et al.*^134^ developed aerogels of ZIF-67/MXene/cellulose through an innovative combination of solution mixing and pyrolysis, resulting in electric/magnetic hybrid carbon aerogels that exhibit superior performance. The intricate porous structure and synergistic effects of electric and magnetic losses enable these aerogels to achieve an EMI SE and an absorption coefficient of 86.7 dB and 0.72, respectively, thus expanding their potential applications in various high-tech domains. The proliferation of electronic devices has heightened concerns regarding the pervasive EM radiation and potential kinetic energy injuries to precision instruments and human health. In response, Sang *et al.*^135^ have proposed a Melamine Sponge/MXene/polyborosiloxane hybrid structure that exhibits remarkable efficient resistance and EMI shielding, demonstrating significant capability in precision electronic systems and wearable protective gear. As demonstrated in Fig. 8a, to explore the practical application of MSM57P0.25 material in wearable protection, the composite was integrated into standard sportswear to develop advanced safety clothing. The resulting MSMP-based protective components demonstrated excellent wearability and adaptability, easily conforming to various body parts such as fingers and wrists, enabling targeted protection during physical activities (see Fig. 8a–d).

Foam-structured polymer composites provide an effective approach for lightweight EMI shielding materials. Their porous architecture promotes multiple reflections, interfacial polarization, and conductive losses, leading to enhanced electromagnetic wave attenuation while maintaining low density. In addition to shielding performance, these materials often exhibit mechanical flexibility, impact absorption, and multifunctional properties, making them suitable for applications in wearable electronics, aerospace, and portable devices.

### 5.3. Rubber

In a study, a rubber composite integrating 2D MXene and EPDM rubber was synthesized, demonstrating a down penetration threshold of 2.7 wt%, electrical conductivity and thermal conductivity of 106 S m^−1^ and 1.57 W m^−1^ K^−1^, respectively, at 6 wt% MXene, alongside impressive EMI shielding capabilities of 48 dB and 52 dB in the X and Ku-bands, respectively, underscoring its potential for diverse applications.^136^ Silicone rubber foam (SiRF) is a valuable material full of porosity used across various domains, yet its inherent flammability poses a considerable challenge. Chen *et al.*, present an innovative and eco-friendly method for enhancing SiRF's flame-retardant properties through the reactive assembly of a minimal quantity of MXene sheets, lead to a composite with superior mechanical adoptability and significant smoke suppression.^137^

The demand for lightweight, highly conductive composites with effective EMI shielding is critical for flexible electronics. Ye *et al.*^138^ presented a novel method for creating flexible Ti_3_C_2_T_*x*_/natural rubber (NR) composites with minimal MXene content, achieving remarkable electrical conductivity and EMI shielding performance while maintaining flexibility and durability under repeated strain. There exists an outstanding demand for advanced EMI shielding compounds characterized by substantial EM wave absorption and minimal reflection, essential for safeguarding emerging communication technologies and wearable electronics. The research conducted by Li *et al.*^139^ stated preparation of silicone rubber/MXene/Fe_3_O_4_ composites according to a unique sandwich-like framework, which significantly enhances EM shielding performance to 55.5 dB, while maintaining durability and tensile strength after extensive bending cycles, indicating promising applications in EM protection for wearable devices. The increasing prevalence of portable electronics necessitates advanced adoptable EMI shielding compounds to address escalating EM wave pollution.

Luo *et al.*^140^ claimed utilizing a vacuum-assisted filtration method to create superb conductive films of Ti_3_C_2_T_*x*_/NR nanocomposite, achieving remarkable electrical conductivity and EMI SE, along with outstanding modification in tensile strength and modulus, thereby indicating their potential utility in future flexible electronics. Lu *et al.*^141^ use a scalable wet spinning technique to fabricate adoptable and conductive fibers consisting from Ti_3_C_2_T_*x*_ MXene aqueous blends and NR.

Recent studies have focused on the development of lightweight and flexible polymer-based composites with enhanced electrical conductivity and effective EMI shielding performance. By incorporating conductive and magnetic nanofillers into elastomeric matrices such as natural rubber and silicone rubber, these materials exhibit improved electrical properties, strong EMI attenuation, and mechanical durability under repeated deformation. In several cases, structural design strategies and scalable fabrication techniques have also enabled improved electromagnetic wave absorption with reduced reflection. Such multifunctional and mechanically adaptable composites show considerable potential for applications in flexible electronics, wearable devices, and advanced electromagnetic protection systems.

### 5.4. Plastic

**Fig. 9:**
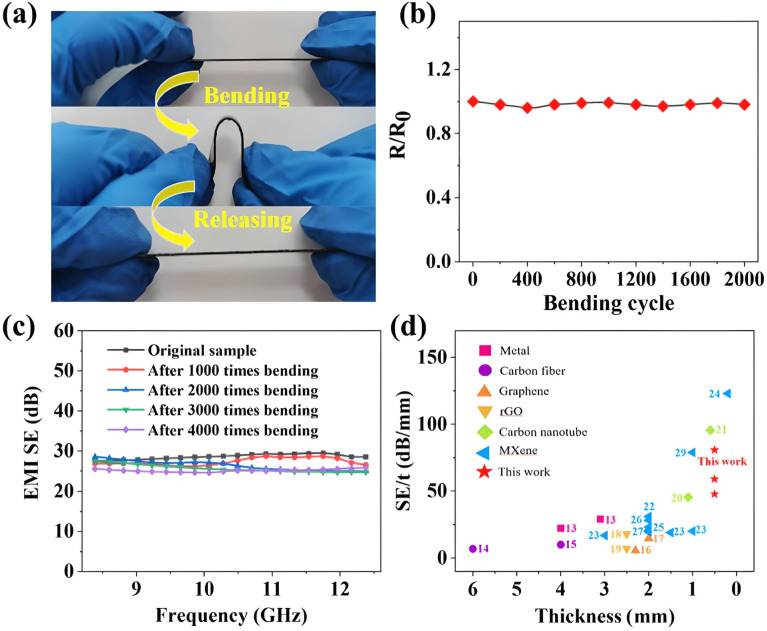
(a) Composite bending functionality and releasing operation of Ti_3_C_2_T_*x*_/CF_f_/TPU-2; (b) *R*/*R*_0_ value (c) EMI shielding value after multiple times bending; (d) comparison data of EMI shielding performance. Reproduced from ref. 142 with permission from Elsevier, copyright 2022.

Designing Ti_3_C_2_T_*x*_-based composites for EMI shielding with superior mechanical characteristics presents significant challenges. Duan *et al.*^142^ successfully developed a robust composite of Ti_3_C_2_T_*x*_/carbon fiber fabric/thermoplastic PUs *via* a straightforward electrohydrodynamic atomization technique, achieving impressive tensile strength and EMI SE while maintaining flexibility and stability after repeated bending cycles. The mechanical flexibility and durability of the Ti_3_C_2_T_*x*_/CF_f_/TPU-2 composite were evaluated through repeated bending and releasing cycles, as illustrated in Fig. 9a. Despite mechanical deformation, the composite maintained stable electrical performance, as indicated Fig. 9b, by the consistent relative resistance (*R*/*R*_0_) values throughout the operation. Furthermore, according to Fig. 9c, the composite retained its EMI SE even after undergoing multiple bending cycles, confirming its robust shielding performance under dynamic conditions. A comparative analysis of the EMI shielding performance with other reported materials is presented in Fig. 9d, highlighting the superior or competitive performance of the Ti_3_C_2_T_*x*_/CF_f_/TPU composite.

Li *et al.*^143^ developed transparent conductive films (TCFs) utilizing Ti_3_C_2_T_*x*_ nanosheets (MXene) on thermoplastic polyurethane (TPU) substrates *via* a straightforward spraying technique, which demonstrated excellent efficacy in some different areas of engineering chemistry like EMI shielding, Joule heating, and photothermal conversion. The optimized TCF exhibited a sheet resistance, transmittance, and an average EMI SE of 119.1 Ω sq^−1^, 43.1%, and 10.9 dB, respectively, showcasing exceptional thermal management capabilities, thus indicating its potential application in various flexible electronic devices. Zhang *et al.*^144^ utilized electrospinning to fabricate thermoplastic polyurethane (TPU) nanofiber membrane, subsequently enhancing its performance by synthesizing poly-dopamine (PDA) coatings and incorporating MXene, resulting in a composite labeled MPT. This innovative MPT nanofiber membrane exhibits exceptional EMI shielding capabilities and efficient strain sensing, indicating its potential application in advanced wearable medical technologies. Sang *et al.*^145^ prepared composite of PTFE/MXene/PI as a multi-functional soft electrothermal actuator *via* a rapid “cutting and sticking” technique, resulting in a robust, hydrophobic structure that exhibits exceptional EMI shielding with a SE_T_ value up to 44 dB because of the excellent conductivity of the MXene film. This actuator demonstrates significant bending capabilities and thermal responsiveness, allowing for various configurations, thus showcasing its potential applications in intelligent EMI shielding systems and soft robotics. Recent studies show that plastic-based conductive composites can achieve effective EMI shielding while maintaining mechanical flexibility and durability. Through fabrication methods such as spraying, electrospinning, and atomization, conductive networks can be integrated into thermoplastic polymers to provide stable electrical performance even under repeated bending. In addition to electromagnetic attenuation, these materials often exhibit multifunctional properties such as electrothermal response, transparency, and strain sensing, making them promising for flexible electronics and wearable devices.

Direct comparison of EMI SE values reported in different studies should be approached with caution. Parameters such as sample thickness, filler loading, testing frequency range, and measurement methods can significantly influence the measured SE values. Therefore, standardized evaluation protocols are essential for enabling reliable comparison across different studies. In addition to absolute SE, normalized parameters such as SSE and thickness-normalized SSE/t are increasingly important for evaluating the practical performance of EMI shielding materials. These parameters provide more reliable comparisons by considering material density and sample thickness, particularly for lightweight polymer nanocomposites. However, the absence of standardized testing and reporting protocols still limits direct comparison among different studies.

## 6. Conclusions and future prospects

Conductive polymeric nanocomposites have emerged as promising materials for next-generation EMI shielding owing to their lightweight nature, mechanical flexibility, corrosion resistance, and tunable electrical properties. Compared with conventional metal-based shields, these materials offer greater design versatility and compatibility with modern electronic systems requiring miniaturization, portability, and multifunctionality. The incorporation of conductive nanofillers such as carbon nanotubes, graphene, MXenes, metal nanowires, and metallic nanoparticles into polymer matrices enables the formation of conductive networks capable of attenuating electromagnetic radiation through reflection, absorption, and interfacial polarization mechanisms. Recent progress in nanofiller engineering, surface functionalization, and fabrication strategies has significantly improved filler dispersion, interfacial compatibility, electrical conductivity, and overall shielding effectiveness. In particular, hybrid filler systems and porous or multilayer structures have demonstrated enhanced absorption-dominated shielding behavior while maintaining desirable mechanical properties. Furthermore, the compatibility of polymeric nanocomposites with scalable processing techniques such as extrusion, injection molding, and additive manufacturing provides strong potential for large-scale industrial production and integration into flexible and wearable electronic devices.

Despite these advancements, several challenges still limit the practical implementation of these materials. Achieving uniform nanofiller dispersion while preventing aggregation remains a major issue, especially at high filler loadings. In addition, balancing electrical conductivity, mechanical flexibility, thermal stability, and long-term durability continue to be a critical design challenge. Conductive polymer-based systems, although highly effective for EMI shielding, are particularly susceptible to oxidation and degradation under humid and oxygen-rich environments, which can gradually reduce conductivity and shielding performance.

Future research should focus on the design of multifunctional nanocomposites with improved structural stability, enhanced absorption-dominated shielding, and integrated functionalities such as thermal management, sensing, self-healing, and energy storage. Strategies involving hierarchical hybrid fillers, interface engineering, and data-driven material optimization may further improve shielding performance while reducing material cost and processing complexity. Continued advances in scalable fabrication, durability enhancement, and predictive materials design are expected to accelerate the practical application of conductive polymeric nanocomposites in wearable electronics, aerospace systems, telecommunications, and other emerging technologies.

## Conflicts of interest

The authors declared no potential conflicts of interest with respect to the research, authorship, and/or publication of this article.

## Data Availability

No primary research results, software or code have been included and no new data were generated or analysed as part of this review.
